# Actions of *Inonotus obliquus* against Hyperuricemia through XOD and Bioactives Screened by Molecular Modeling

**DOI:** 10.3390/ijms19103222

**Published:** 2018-10-18

**Authors:** Tianqiao Yong, Shaodan Chen, Danling Liang, Dan Zuo, Xue Diao, Chenling Deng, Yuning Wu, Huiping Hu, Yizhen Xie, Diling Chen

**Affiliations:** 1 State Key Laboratory of Applied Microbiology Southern China, Guangdong Provincial Key Laboratory of Microbial Culture Collection and Application and Guangdong Open Laboratory of Applied Microbiology, Guangdong Institute of Microbiology, Guangzhou 510070, China; chenshaodan@126.com (S.C.); liangdanling@hotmail.com (D.L.); xuediao92@126.com (X.D.); 15820115385@163.com (C.D.); qaz_wuyuning@163.com (Y.W.); huhp@gdim.cn (H.H.); diling1983@163.com (D.C.); 2 Guangdong Yuewei Edible Fungi Technology Co., Guangzhou 510663, China; 3 College of Chinese Materia Medica, Guangzhou University of Traditional Chinese Medicine, Guangzhou 510070, China; 4 Guangzhou Institutes of Biomedicine and Health, Chinese Academy of Sciences, Guangzhou 510530, China; zuo_dan@gibh.ac.cn

**Keywords:** *Inonotus obliquus*, hyperuricemia, xanthine oxidase, uric acid transporter 1, betulin

## Abstract

*Inonotus obliquus* is an edible mushroom and also a remedy against various diseases, especially metabolic syndrome. In this paper we report the actions of an ethanol extract of *I. obliquus* (IOE) against hyperuricemia in hyperuricemic mice, and the screen of bioactives. The extract (IOE) was prepared by extracting *I. obliquus* at 65 °C with ethanol, and characterized by HPLC. IOE at low, middle, and high doses reduced serum uric acid (SUA) of hyperuricemic mice (353 μmol/L) to 215, 174, and 152 μmol/L (*p* < 0.01), respectively, showing similar hypouricemic effectiveness to the positive controls. IOE showed a non-toxic impact on kidney and liver functions. Of note, IOE suppressed xanthine oxidase (XOD) activity in serum and liver, and also down-regulated renal uric acid transporter 1 (URAT1). Four compounds hit highly against XOD in molecular docking. Overall, the four compounds all occupied the active tunnel, which may inhibit the substrate from entering. The IC_50_ of betulin was assayed at 121.10 ± 4.57 μM, which was near to that of allopurinol (148.10 ± 5.27 μM). Betulin may be one of the anti-hyperuricemia bioactives in *I. obliquus*.

## 1. Introduction

*Inonotus obliquus* belongs to the Hymenochaetaceae family and is a white-rot fungus parasitizing on birches throughout Eurasia [1]. It is an edible mushroom, and also a traditional remedy used for curing various diseases since the 12th century, such as cerebrovascular diseases, diabetes, and gastrointestinal diseases [2]. Modernly, it was verified that it was effective against hyperglycemia [3], cancer [4], inflammation [5], and especially regulation of metabolism [1]. Bioactives, such as triterpenes, polysaccharides, polyphenols, and melanin, have been isolated and characterized in *I. obliquus*, and are responsible for antitumor activities [6,7,8], anti-inflammatory ability [5], antioxidant effects [9], hypoglycemic ability [3], immunomodulatory activity [10], and antimutagenic properties [11]. Additionally, its hypouricemic effects were mentioned simply [12].

However, its bioactive constituents and mechanisms against hyperuricemia were not involved. In this paper, we report the preparation of an ethanol extract of *I. obliquus* (IOE), and the investigation of its effects and mechanisms against hyperuricemia in hyperuricemic mice. To understand its mechanisms, xanthine oxidase (XOD) activity, renal organic anion transporter 1 (OAT1), glucose transporter 9 (GLUT9), uric acid transporter 1 (URAT1), and gastrointestinal concentrate nucleoside transporter 2 (CNT2) were examined by RT-PCR and western blot. Additionally, its impacts on internal organs were recorded to evaluate the general toxicity. Computational screening was performed by exploiting our in-house compound database of *I. obliquus* to pick the bioactives. Obtained compounds were assayed to confirm their inhibitory effects on xanthine oxidase (XOD) in vivo.

## 2. Results

**Figure 1:**
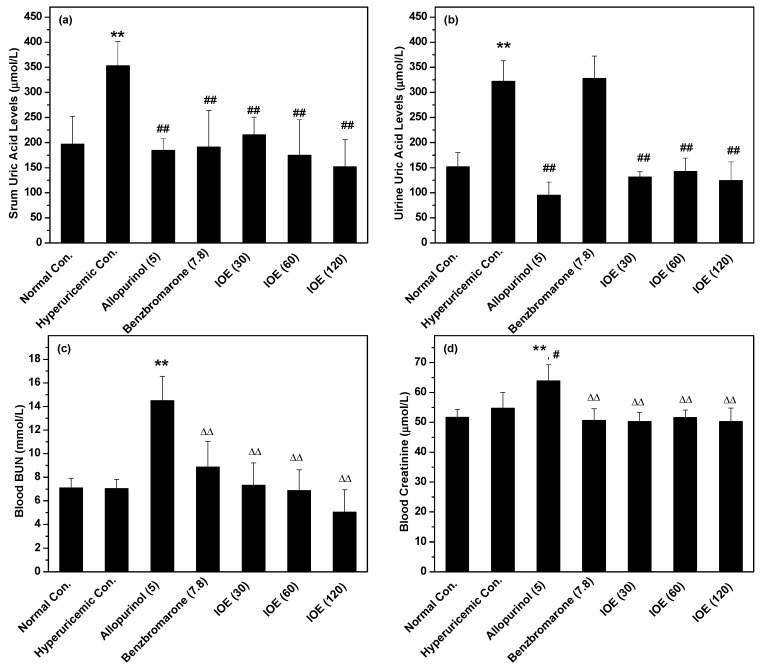
Influences of the ethanol extract of *I. obliquus* (IOE) on (**a**) serum uric acid (SUA), (**b**) urine uric acid (UUA), (**c**) blood urea nitrogen (BUN), and (d) blood creatinine. The administered dosages are included in brackets with units of mg/kg. ** *p* < 0.01 compared with normal control; ^#^ *p* < 0.05, ^##^ *p* < 0.01, compared with hyperuricemic control; ^ΔΔ^ *p* < 0.01 compared with allopurinol control.

The serum uric acid (SUA) of the hyperuricemic control (353 μmol/L) was elevated dramatically from that of normal mice (normal control, 197 μmol/L, *p* < 0.01), suggesting the success of the established model (Figure 1a). As expected, the two positive drug controls, allopurinol and benzbromarone, decreased SUA of hyperuricemic mice to 184 and 191 μmol/L (*p* < 0.01), respectively. Notably, IOE at low, middle, and high doses reduced SUA to 215, 174, and 152 μmol/L (*p* < 0.01), respectively, showing clear hypouricemic action.

Since SUA is associated directly to urine uric acid (UUA), UUA was recorded (Figure 1b). UUA of the hyperuricemic control was elevated to 322 μmol/L from the normal control (152 μmol/L, *p* < 0.01). UUA of IOE at various doses was decreased to 132, 143, and 125 μmol/L (*p* < 0.01). The hypouricemic effect of IOE may not be raised from elevating uric acid excretion.

Blood urea nitrogen (BUN) was also recorded as it is an important index for renal function. Apparently, allopurinol (14.48 mmol/L) depicted a higher BUN than the normal (7.09 mmol/L) and hyperuricemic (7.02 mmol/L) controls (*p* < 0.01, Figure 1c). However, IOE (7.32, 6.86, and 5.04 mmol/L for three doses) showed no increasing effect on BUN. Blood creatinine was also assayed (Figure 1d), and was used as a supplementary index for renal function. Being consistent with the blood BUN result, blood creatinine for allopurinol (64 μmol/L) was higher than the normal (52 μmol/L) and hyperuricemic (54 μmol/L) controls (*p* < 0.01 and 0.05). However, IOE at various doses showed blood creatinine at 50, 52, and 50 μmol/L, which was close to the normal control. According to the blood BUN and creatinine results, IOE demonstrated little-to-no toxic impact on renal function.

**Figure 2:**
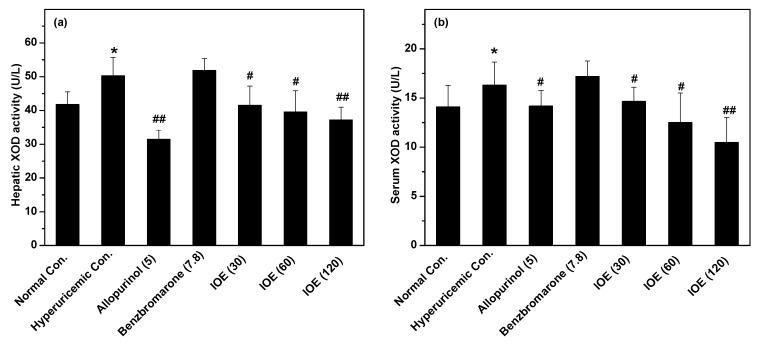
Effects of IOE on (**a**) liver and (**b**) serum xanthine oxidase (XOD) activity. The administered dosages are included in brackets with units of mg/kg. * *p* < 0.05, compared with normal control; ^#^ *p* < 0.05, ^##^ *p* < 0.01, compared with hyperuricemic control.

To illustrate the mechanisms of IOE preventing hyperuricemia, hepatic and serum XOD activities were assayed, because XOD plays a key role in SUA production (Figure 2). For the hyperuricemic control, XOD activity was enhanced to 50.28 and 16.30 U/L (*p* < 0.05) in liver and serum, respectively, in comparison with the normal control in liver (41.76 U/L) and serum (14.10 U/L); the amounts for the intake of hypoxanthine (HX) for the model establishment. Allopurinol, as a marketed XOD inhibitor, reduced XOD activity significantly to 31.43 U/L in liver and 14.18 U/L in serum (*p* < 0.01). Similar to allopurinol, IOE at three doses decreased XOD activity from the hyperuricemic control to 41.50, 39.56, and 37.16 U/L in liver, and 14.65, 12.50, and 10.47 U/L in serum (*p* < 0.05 or 0.01), suggesting XOD inhibition may be one of the mechanisms of *I. obliquus*’ hypouricemic effects.

**Figure 3:**
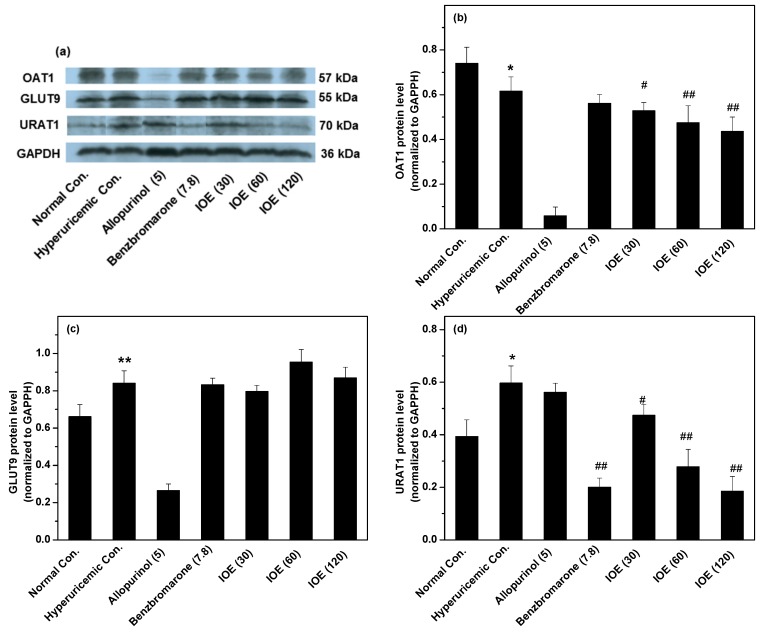
Actions of IOE on renal transporters: Organic anion transporter 1 (OAT1) (**a**,**b**); glucose transporter 9 (GLUT9) (**a**,**c**); and uric acid transporter 1 (URAT1) (**a**,**d**). The administered dosages are included in brackets with units of mg/kg. * *p* < 0.05, ** *p* < 0.01, compared with normal control; ^#^ *p* < 0.05, ^##^ *p* < 0.01, compared with hyperuricemic control.

In order to examine the effects of IOE on the key renal transporters for hyperuricemia, three transporters were assayed by Western blot analysis (Figure 3). However, IOE demonstrated some down-regulations on OAT1. In addition, IOE did not suppress GLUT9. Thus, IOE’s hypouricemic effect was not attributed to its regulations of OAT1 and GLUT9. However, URAT1 were suppressed by IOE in comparison with the hyperuricemic control (*p* < 0.05 or 0.01), suggesting URAT1 suppression may be one of the mechanisms of IOE’s hypouricemic effect.

**Figure 4:**
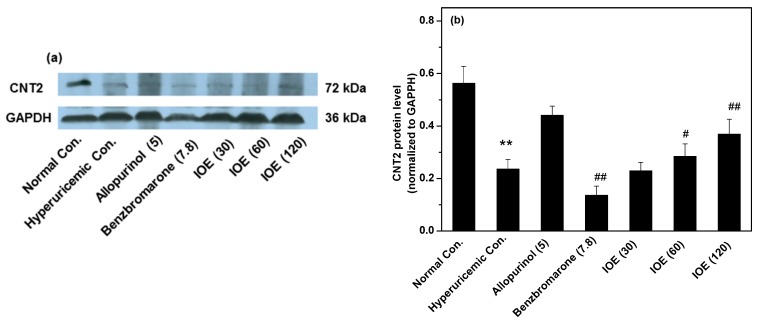
Influence of IOE on gastrointestinal concentrative nucleoside transporter 2 (CNT2). Intestine cortex was extracted for Western blot analysis of CNT2 (**a**,**b**). The administered dosages are included in brackets with units of mg/kg. ** *p* < 0.01 compared with normal control; ^#^ *p* < 0.05, ^##^ *p* < 0.01, in comparison with hyperuricemic control.

Since purine absorption was performed in the gastrointestinal tract, the CNT2 lining of it was analyzed (Figure 4), which played a central role in purine absorption. Obviously, CNT2 was enhanced by middle and high doses of IOE. IOE’s hypouricemic effect was not due to its regulation of CNT2.

**Figure 5:**
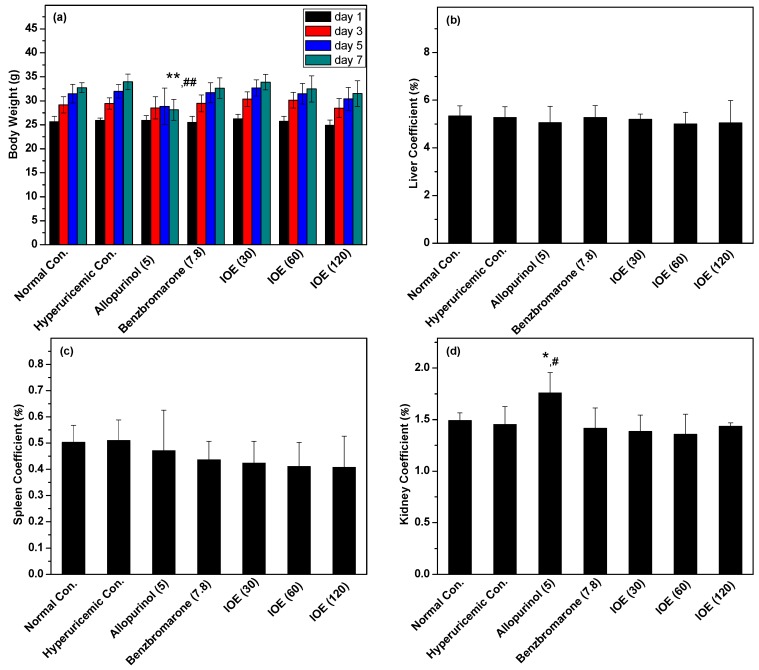
Effects of IOE on (**a**) body weight, (**b**) liver coefficients, (**c**) kidney coefficients, and (**d**) spleen coefficients in mice. The administered dosages are included in brackets with units of mg/kg. * *p* < 0.01, ** *p* < 0.01, compared with normal control; ^#^ *p* < 0.05, ^##^ *p* < 0.01, in comparison with hyperuricemic control.

Body weights and inner organ coefficients were recorded to illustrate the general toxicity of IOE (Figure 5). Apparently, the positive control drug allopurinol inhibited normal body weight growth (*p* < 0.01) of hyperuricemic mice. This negative action was not caught by IOE at various doses. For liver and spleen coefficients, no significant difference was noted between IOE groups and normal groups. However, the kidney coefficient of allopurinol was higher than others (*p* < 0.01).

**Figure 6:**
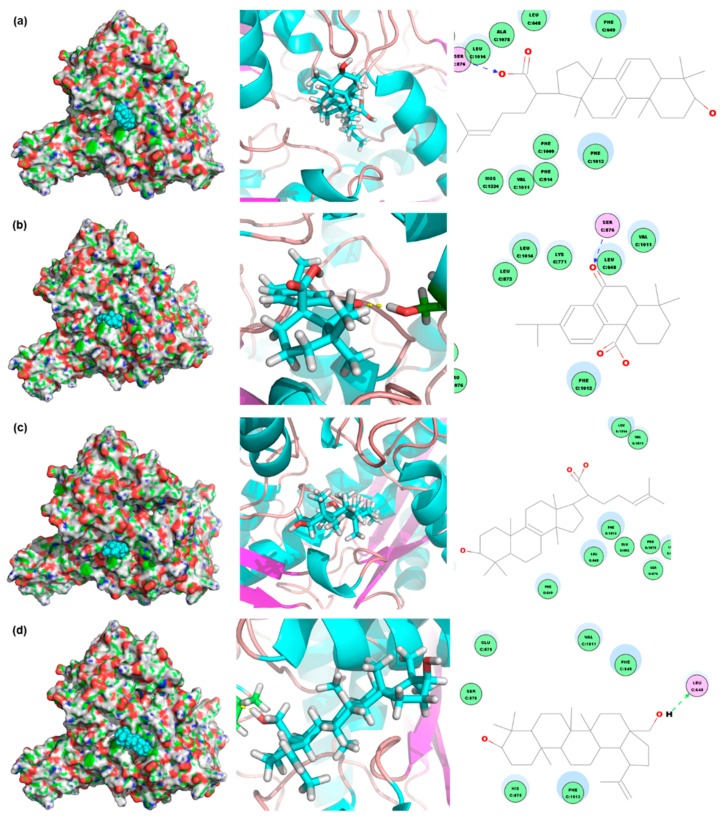
The four top-ranked compounds screened from our in-house database of *I. obliquus* by molecular docking: (**a**) 3β-hydroxylanosta-7,9(11),24-trien-21-oic acid; (**b**) inonotusic acid; (**c**) trametenolic acid; (**d**) betulin.

In order to identify the potential compounds in *I. obliquus* that work against hyperuricemia, we selected XOD (PDBID: 1FIQ) as the target to screen the in-house database of *I. obliquus* built by us. As depicted in Figure 6, four compounds were highly hit. Overall, the four compounds all occupied the active tunnel for substrate entering of XOD, which may inhibit the substrate from entering. Of the four, 3β-hydroxylanosta-7,9(11),24-trien-21-oic acid had the highest Gold score of 6.27 (Figure 6a). It formed a hydrogen bond with SER876 via the oxygen atom of a carboxyl group attached on the aliphatic moiety. Inonotusic acid was ranked second, with a Gold score of 5.40 (Figure 6b). It also bonded with SER876 through a hydrogen bond via the oxygen atom of a carbonyl group attached on the middle cyclic moiety of the tri-cyclic molecule. No polar interaction was observed for trametenolic acid (Gold score: 3.42; Figure 6c). Betulin had the fourth rank with a Gold score of 3.08 (Figure 6d). It interacted with the XOD target through a hydrogen bond to LEU648 via a hydrogen atom of a hydroxyl group attached to the penta-cyclic ring of the molecule.

**Figure 7:**
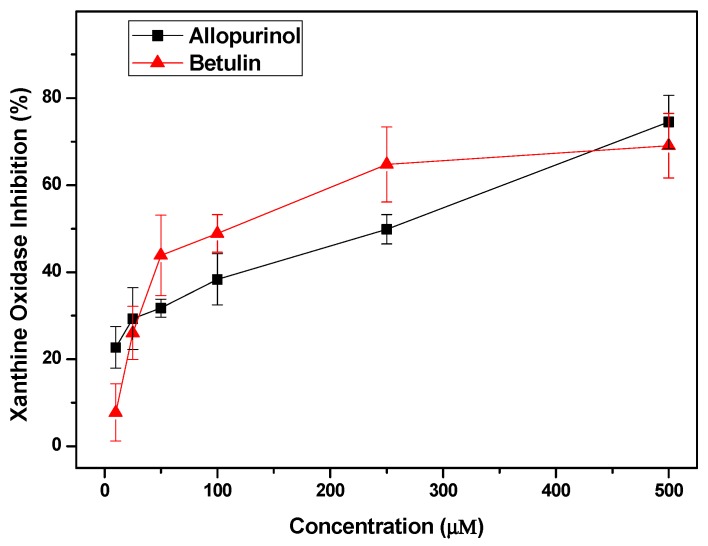
XOD inhibition by betulin.

To verify the hypouricemic effects of the compounds screened in vitro, we performed XOD inhibition assaying using betulin due to its high ranking and availability in our group, and allopurinol as a positive control. As shown in Figure 7, the activity of XOD was inhibited by botulin in a concentration-dependent manner. The IC_50_ of betulin (121.10 ± 4.57 μM) is lower than allopurinol (148.10 ± 5.27 μM). The results indicated that betulin had the ability to inhibit XOD, and it may be an anti-hyperuricemia bioactive compound.

## 3. Discussion

*I. obliquus* has been used as a remedy to cure various diseases, especially metabolic syndrome, based on a long tradition of empirical use since the 12th century. However, there remains a lack of information about its efficacy, bioactives, and mechanisms in the literature. In this paper, we provided the evidence for its new industrial use against hyperuricemia, which we have endeavored to do for several years [13,14]. The ethanol extract of *I. obliquus* (IOE) exhibited outstanding hypouricemic actions, causing remarkable declines in SUA in hyperuricemic mice through suppressing XOD and renal URAT1, providing important information for its good development and utilization prospects. Additionally, several bioactives against XOD [15] were screened out by molecular docking [16,17] and then confirmed in vitro.

Hyperuricemia is characterized by a high SUA, which induces gout directly, and is significantly correlated with cardiovascular diseases, hyperlipidemia, and hypertension. For decades its prevalence has been climbing higher and higher. In terms of this, aberrations of uric acid production and excretion may induce hyperuricemia. Long-term maintenance of hyperuricemia may induce sodium urate crystallization in joints and kidneys leading to gout, which is characterized by repeated inflammation and acute pain. This threatens the quality of life of patients heavily. Therefore, the development and discovery of novel agents against it is drawing increasing attention. *I. obliquus* was exploited as a traditional medicine for metabolic diseases, including diabetes and obesity. We hypothesized that hyperuricemia, as a kind of metabolic disease, may be cured or prevented by it.

SUA was focused on as the main marker for hyperuricemia research. On the other hand, the hyperuricemic model is the key for hyperuricemia-associated disease research. In this study, the clearly elevated SUA of the hyperuricemic control compared with the normal control validated the success of the established hyperuricemic model. Of note, IOE at various doses reduced SUA significantly in hyperuricemic mice, suggesting IOE’s hypouricemic effects. Furthermore, IOE showed some similar effectiveness against allopurinol and benzbromarone, demonstrating promising value.

SUA homeostasis is influenced by UUA directly, since it is excreted by the kidney in the form of UUA. For this hyperuricemic model, UUA increased significantly because of the intake of 500 mg/kg HX (hypoxanthine) for model establishment, which was converted into uric acid in the liver and serum by XOD, and then distributed into the blood. UUA of the allopurinol control was decreased heavily because allopurinol served as a XOD inhibitor. But benzbromarone acted as a uricosuric, so it promoted UUA. IOE at various doses reduced UUA, which was similar to the XOD inhibitor allopurinol. Therefore, IOE did not serve as a uricosuric agent, but may be a XOD inhibitor. Renal dysfunction is frequently accompanied by serum BUN and creatinine elevation in PO (potassium oxonate) treated mice. However, the BUN and creatinine were not significantly elevated due to the low usage of PO (just 100 mg/kg). Obviously, IOE at three doses did not show negative effects, suggesting non-toxicity on renal function.

XOD is a single-gene product [15,18] and the key enzyme involved in uric acid production, which is the final metabolite of purines. Inhibition of this pathway results in decreased uric acid production and loading of uric acid in joints. Accordingly, XOD is a validated target for therapeutic treatment of gout and other conditions associated with hyperuricemia [19]. Several XOD inhibitors are already present on the market [20,21]. To illustrate its mechanisms for hyperuricemia prevention, XOD activities in hepatic and serum were examined, and suppressive effects of IOE were observed against XOD in liver and serum. Therefore, XOD inhibition was one of the mechanisms of *I. obliquus*’ hypouricemic effects.

In healthy humans, kidneys play a primary role in controlling SUA, where SUA is secreted out of the body in the form of UUA, and then reabsorbed into the body by nephron cells. The cells lining the nephron contain specific transporters which dominate uric acid excretion and reabsorption. The basolateral surface contains OAT1 [22], which transports uric acid from blood to nephron cells. The basolateral surface also contains GLUT9 [23], which has the function of uric absorption from nephron cells to blood. The apical surface contains URAT1 [24], which has the function of uric acid reabsorption from urine to nephron cells. Generally, increased OAT1, and decreased GLUT9 and URAT1, accelerate uric acid excretion to urine. However, OAT1 was suppressed in this study. Therefore, IOE influence on it did not participate in uric acid excretion enhancement. URAT1 was also suppressed, and may intervene in uric acid excretion improvement. CNT2 is the preferred purine nucleoside transporter because it transports purines and uridines into blood from food intake. Thus, CNT2 is likely to be the key transporter for the uptake of purine nucleosides in the gastrointestinal tract, which may induce hyperuricemia. Hence, its inhibitors would suppress the increase in SUA derived from dietary purines. However, CNT2 was not suppressed by IOE.

Organ weights are utilized to evaluate general toxicities conventionally [25]. Liver coefficients may be more appropriate to reflect the status of liver function [26]. Usually, it may be increased by potent hepatic enzyme-inducing compounds. In this study, IOE did not enhance liver coefficients, suggesting no toxicity in the liver. Renal coefficients mark the tubular hypertrophy, chronic progressive nephropathy, or renal toxicity [27]. Contrary to the toxic effect of allopurinol on kidneys, IOE showed no toxicity in kidneys. Therefore, it is clear that *I. obliquus* has considerable potential as a safe agent for applications in functional foods or therapeutics against hyperuricemia.

Virtual screening is usually exploited to studying bioactives of medicinal plants or mushrooms [13]. Therefore, molecular docking was conducted to identify the potential anti-hyperuricemia bioactives in *I. obliquus* in this paper. XOD was selected as the target since IOE showed XOD inhibitory effects in vivo in hyperuricemic mice. Additionally, a database of compounds in *I. obliquus* was established by accumulating the structures reported in literature. Results showed several poses with good scores. The top four compounds were identified and listed, and they all occupied the active tunnel, which may inhibit the substrate from entering. To verify the predicted result, XOD inhibition assay was performed. From this, Betulin demonstrated a significant XOD inhibition effect, and it may be an anti-hyperuricemia bioactive compound.

In summary, the ethanol extract of the edible and medicinal mushroom *I. obliquus* (IOE) at low, middle, and high doses reduced SUA to 215, 174, and 152 μmol/L (*p* < 0.05), respectively, from 353 μmol/L of hyperuricemic control, showing comparable hypouricemic effectiveness to allopurinol and benzbromarone. IOE demonstrated a non-toxic action to renal function according to BUN, creatinine, and kidney coefficient results. IOE at three doses decreased XOD activity in hepatic and serum, suggesting XOD inhibition may be one of the mechanisms of *I. obliquus*’ hypouricemic effect. Thus, in order to identify the potential compounds in *I. obliquus* against hyperuricemia, we selected XOD as the target to screen the in-house database of *I. obliquus* built by us, wherein four compounds were hit highly. Overall, the four compounds all occupied the active tunnel, which may inhibit the substrate from entering. To verify the hypouricemic effects of the compounds screened in vitro, we performed XOD inhibitory assaying, using betulin due to its high ranking and availability, and allopurinol as the control. The IC_50_ of betulin was 121.10 ± 4.57 μM, which was lower than that of allopurinol (148.10 ± 5.27 μM). Betulin may be one of the anti-hyperuricemia bioactives in *I. obliquus*.

## 4. Materials and Methods

### 4.1. Materials

Potassium oxonate (PO), hypoxanthine (HX), allopurinol, benzbromarone, and betulin were obtained from Alladin Reagent Co. (Shanghai, China). Xanthine and XOD for in vitro XOD activity assaying were obtained from Sigma–Aldrich LLC. (St. Louis, MI, USA). Guangdong Yuewei Edible Fungi Technology Co. (Guangzhou, China) supplied and identified *I. obliquus* samples, and a voucher specimen (YW20170703-IO) was stored in the herbarium of the Guangdong Institute of Microbiology. One hundred grams of *I. obliquus* was ground and then extracted with 2 L ethanol at 65 °C three times to afford IOE (yield 4.87 g; 4.8%). Its HPLC fingerprints (Figure S1), standard chemical HPLC chromatograph (Figure S2), and HPLC conditions are offered in the supplementary materials. PO and HX for model establishment; allopurinol and benzbromarone as positive drugs, and IOE as drugs at various concentrations; were dissolved in water to afford solutions at the proper concentration for the model establishment and drug administration, respectively. Uric acid, in vivo XOD activity, and blood urea nitrogen (BUN) and creatinine kits were purchased from Nanjing Jian Cheng Bioengineering Institute (Nanjing, China), R & D System Inc. (Minneapolis, MN, USA), and Mindray Medical Corp. (Shenzhen, China), respectively.

### 4.2. Animals, Models, and Drug Administration

SPF Kunming mice (male) with a weight of 20 ± 2 g were supplied by the Guangdong Provincial Medical Laboratory Animal Centre (Guangzhou, China). The animal experiment was verified and performed by and in the Guangdong Institute of Microbiology (approved ID: GT-IACUC20170721-1; Guangzhou, China). Specifically, after adapting for 7 days, animals were separated (*n* = 10) into normal, hyperuricemia, allopurinol, and benzbromarone controls, and drug groups with IOE, where allopurinol (5 mg/kg) and benzbromarone (7.8 mg/kg) served as positive controls, and IOE (30, 60, 120mg/kg) as drug groups. The protocol reported by us [13] was utilized to establish models for all groups except the normal control. Specifically, HX (500 mg/kg) and PO (100 mg/kg) were given to enhance serum uric acid (SUA). The administration of the drugs allopurinol (5 mg/kg), benzbromarone (7.8 mg/kg), and IOE at 30, 60, and 120 mg/kg, was conducted at 1 h after the model establishment.

### 4.3. Physiological Parameters

SUA, urine uric acid (UUA), blood urea nitrogen (BUN), and creatinine were assayed according to the manufacturers’ instructions using purchased kits on an Automatic Biochemistry Analyzer (7020 HITACHI, Chiyoda, Tokyo, Japan). Body weights and internal organ coefficients (organ-to-body weight ratios) were recorded to evaluate the general toxicity of IOE.

### 4.4. In vivo XOD Activity, and Renal and Gastrointestinal Tract Transporters

The in vivo XOD activity of liver and blood was assayed using the ELISA Kit described above (R&D System Inc., Minneapolis, MN, USA) according to the manufacturer’s instructions, with the liver and blood samples collected from mice after the animal experiment. Hyperuricemia correlated key transporters, such as renal transporters (OAT1, GLUT9, and URAT1) and gastrointestinal transporters (CNT2), were assayed by Western blot to examine their mechanisms against hyperuricemia with antibodies described previously [14].

### 4.5. Compound Database Establishment and Virtual Screening

A chemical information database including 52 compounds (Figure S3) isolated from *I. obliquus* was established by retrieving data from Internet search engines, such as the Chemical Abstracts Service (CAS) database, Web of Science, and ChemSpider. They were then energy minimized using CHARMm force fields, and thereafter exploited as ligands. XOD structure (PDBID: 1FIQ) [15] was downloaded from the RCSB database, which was used frequently for XOD inhibitor screening. The active pocket was defined as a sphere of 10 Å in diameter, which located salicylic acid as the center. Molecular docking was performed using Gold software [16] and the top poses were picked.

### 4.6. Confirming the Anti-Hyperuricemic Effects of Screened Compounds by In Vitro Inhibitory Experiments

The inhibition of screened compounds on the formation of uric acid was measured using xanthine as the substrate on a ShimadzuUV-2450 spectrophotometer (Shimadzu, Japan) equipped with a 1.0 cm path length cell. A series of 2.0 mL reaction mixtures consisting of various amounts of screened compounds and a fixed concentration of XOD (7.5 × 10^−8^ mol L^−1^) were prepared by solving and mixing with PBS (phosphate buffer saline, pH = 7.4), and incubated for 0.5 h at 37 °C. Then the reaction was started by adding substrate xanthine (final concentration 5.0 × 10^−5^ mol L^−1^), and the XOD activity was assayed spectrophotometrically by monitoring uric acid production at a wavelength of 290 nm [17]. The inhibition (%) = 100% × [1 − (slope of reaction kinetics equation obtained by reaction with inhibitor)/(slope of reaction kinetics equation obtained by reaction without inhibitor)]. PBS and allopurinol were used as negative and positive controls, respectively.

## Supplementary Materials

Supplementary materials can be found at http://www.mdpi.com/1422-0067/19/10/3222/s1.

## Abbreviations

SUA	serum uric acid
XOD	xanthine oxidase
PBS	phosphate buffer saline
BUN	blood urine nitrogen
OAT1	organic anion transporter 1
UART1	uric acid transporter 1
GLUT9	glucose transporter 9
CNT2	gastrointestinal concentrative nucleoside transporter 2
PO	potassium oxonate
HX	hypoxanthine
IOE	ethanol extract of *Inonotus obliquus*

## References

[1] Shikov A.N., Pozharitskaya O.N., Makarov V.G., Wagner H., Verpoorte R., Heinrich M. (2014). Medicinal plants of the russian pharmacopoeia; their history and applications. J. Ethnopharmacol..

[2] Choi S.Y., Hur S.J., An C.S., Jeon Y.H., Jeoung Y.J., Bak J.P., Lim B.O. (2010). Anti-inflammatory effects of *Inonotus obliquus* in colitis induced by dextran sodium sulfate. J. Biomed. Biotechnol..

[3] Ying Y.M., Zhang L.Y., Zhang X., Bai H.B., Liang D.E., Ma L.F., Shan W.G., Zhan Z.J. (2014). Terpenoids with alpha-glucosidase inhibitory activity from the submerged culture of *Inonotus obliquus*. Phytochemistry.

[4] Liu C., Zhao C., Pan H.H., Kang J., Yu X.T., Wang H.Q., Li B.M., Xie Y.Z., Chen R.Y. (2014). Chemical constituents from *Inonotus obliquus* and their biological activities. J. Nat. Prod..

[5] Ma L., Chen H., Dong P., Lu X. (2013). Anti-inflammatory and anticancer activities of extracts and compounds from the mushroom *Inonotus obliquus*. Food Chem..

[6] Zhao F., Mai Q., Ma J., Xu M., Wang X., Cui T., Qiu F., Han G. (2015). Triterpenoids from *Inonotus obliquus* and their antitumor activities. Fitoterapia.

[7] Handa N., Yamada T., Tanaka R. (2010). An unusual lanostane-type triterpenoid, spiroinonotsuoxodiol, and other triterpenoids from *Inonotus obliquus*. Phytochemistry.

[8] Nomura M., Takahashi T., Uesugi A., Tanaka R., Kobayashi S. (2008). Inotodiol, a lanostane triterpenoid, from *Inonotus obliquus* inhibits cell proliferation through caspase-3-dependent apoptosis. Anticancer Res..

[9] Glamoclija J., Ciric A., Nikolic M., Fernandes A., Barros L., Calhelha R.C., Ferreira I.C., Sokovic M., van Griensven L.J. (2015). Chemical characterization and biological activity of chaga (*Inonotus Obliquus*), a medicinal “mushroom”. J. Ethnopharmacol..

[10] Fan L., Ding S., Ai L., Deng K. (2012). Antitumor and immunomodulatory activity of water-soluble polysaccharide from *Inonotus obliquus*. Carbohydr. Polym..

[11] Ham S.S., Kim S.H., Moon S.Y., Chung M.J., Cui C.B., Han E.K., Chung C.K., Choe M. (2009). Antimutagenic effects of subfractions of chaga mushroom (*Inonotus Obliquus*) extract. Mutat. Res..

[12] Zhang Z., Sun W., Li W., Wang Y., Wang C. (2010). Study on effect of the extract of *Inonotus obliquus* on rats hyperuricemia. Food Sci. Technol..

[13] Yong T., Zhang M., Chen D., Shuai O., Chen S., Su J., Jiao C., Feng D., Xie Y. (2016). Actions of water extract from cordyceps militaris in hyperuricemic mice induced by potassium oxonate combined with hypoxanthine. J. Ethnopharmacol..

[14] Yong T., Chen S., Xie Y., Chen D., Su J., Shuai O., Jiao C., Zuo D. (2017). Hypouricemic effects of ganoderma applanatum in hyperuricemia mice through oat1 and glut9. Front. Pharmacol..

[15] Cao H., Hall J., Hille R. (2011). X-ray crystal structure of arsenite-inhibited xanthine oxidase: Μ-sulfido,μ-oxo double bridge between molybdenum and arsenic in the active site. J. Am. Chem. Soc..

[16] Verdonk M.L., Cole J.C., Hartshorn M.J., Murray C.W., Taylor R.D. (2003). Improved protein-ligand docking using gold. Proteins.

[17] Chu Y.H., Chen C.J., Wu S.H., Hsieh J.F. (2014). Inhibition of xanthine oxidase by rhodiola crenulata extracts and their phytochemicals. J. Agric. Food Chem..

[18] Prusis P., Dambrova M., Andrianov V., Rozhkov E., Semenikhina V., Piskunova I., Ongwae E., Lundstedt T., Kalvinsh I., Wikberg J.E.S. (2004). Synthesis and quantitative structure−activity relationship of hydrazones of n-amino-n′-hydroxyguanidine as electron acceptors for xanthine oxidase. J. Med. Chem..

[19] Enroth C., Eger B.T., Okamoto K., Nishino T., Nishino T., Pai E.F. (2000). Crystal structures of bovine milk xanthine dehydrogenase and xanthine oxidase: Structure-based mechanism of conversion. Proc. Natl. Acad. Sci. USA.

[20] Halevy S., Ghislain P.-D., Mockenhaupt M., Fagot J.-P., Bouwes Bavinck J.N., Sidoroff A., Naldi L., Dunant A., Viboud C., Roujeau J.-C. (2008). Allopurinol is the most common cause of stevens-johnson syndrome and toxic epidermal necrolysis in europe and israel. J. Am. Acad. Dermatol..

[21] Becker M.A., Schumacher H.R., Espinoza L.R., Wells A.F., MacDonald P., Lloyd E., Lademacher C. (2010). The urate-lowering efficacy and safety of febuxostat in the treatment of the hyperuricemia of gout: The confirms trial. Arthritis Res. Ther..

[22] Eraly S.A., Vallon V., Rieg T., Gangoiti J.A., Wikoff W.R., Siuzdak G., Barshop B.A., Nigam S.K. (2008). Multiple organic anion transporters contribute to net renal excretion of uric acid. Physiol. Genom..

[23] Liu S., Yuan Y., Zhou Y., Zhao M., Chen Y., Cheng J., Lu Y., Liu J. (2017). Phloretin attenuates hyperuricemia-induced endothelial dysfunction through co-inhibiting inflammation and glut9-mediated uric acid uptake. J. Cell. Mol. Med..

[24] Enomoto A., Kimura H., Chairoungdua A., Shigeta Y., Jutabha P., Cha S.H., Hosoyamada M., Takeda M., Sekine T., Igarashi T. (2002). Molecular identification of a renal urate anion exchanger that regulates blood urate levels. Nature.

[25] Michael B., Yano B., Sellers R.S., Perry R., Morton D., Roome N., Johnson J.K., Schafer K. (2007). Evaluation of organ weights for rodent and non-rodent toxicity studies: A review of regulatory guidelines and a survey of current practices. Toxicol. Pathol..

[26] Bailey S.A., Zidell R.H., Perry R.W. (2004). Relationships between organ weight and body/brain weight in the rat: What is the best analytical endpoint?. Toxicol. Pathol..

[27] Greaves P. (2000). IX-Urinary Tract. Histopathology of Preclinical Toxicity Studies.

