# Supplementary material for: Actions of Inonotus obliquus against Hyperuricemia through XOD and Bioactives Screened by Molecular Modeling
Source: Int J Mol Sci. 2018 Oct 18;19(10):3222. doi: 10.3390/ijms19103222 (PMC6214139; doi:10.3390/ijms19103222)
Supplement: Supplementary file 1 [file ijms-19-03222-s001.pdf]

Supporting Information

Actions of a Forest Corp *Inonotus obliquus* against Hyperuricemia through XOD and its Effective Bioactives Screened by Molecular Modeling and Confirmed *in vitro*

Tianqiao Yong [a, b, *], Shaodan Chen [a, b], Danling Liang [a, b, c], Dan Zuo [d], Xue Diao [a, b], Chenling Deng [a, b], Yuning Wu [a, b], Huiping Hu [a], Yizhen Xie [a, b], Diling Chen [a]

[a] State Key Laboratory of Applied Microbiology Southern China, Guangdong Provincial Key Laboratory of Microbial Culture Collection and Application and Guangdong Open Laboratory of Applied Microbiology, Guangdong Institute of Microbiology, Guangzhou 510070, China

[b] Guangdong Yuewei Edible Fungi Technology Co., Guangzhou 510663, China

[c] College of Chinese Materia Medica, Guangzhou University of Traditional Chinese Medicine, Guangzhou, China

[d] Guangzhou Institutes of Biomedicine and Health, Chinese Academy of Sciences, Guangzhou 510530, China

***Corresponding author:** Tianqiao Yong, Fax: +86-20-32059602, E-mail: tianqiao@mail.ustc.edu.cn

Experimental

1. HPLC Conditions

Chromatography was performed on Agilent 1200 HPLC with a reversed-phase column (Waters Atlantis T3 RP-C18 column, 5 μm, 250 mm × 4.6 mm) with a flow rate of 1.0 mL/min. Separation was carried out by linear gradient elution with methanol (0.1 % formic acid, A) and water (B) with the following elution procedures: 0 min (10% A)→50 min (100% A)→70 min (100% A)→71 min (10% A)→83 min (10% A). The detection wavelength was set at 280 nm, and the column temperature was kept at 30 °C. The loading volume was 10 μL.

2. Compound Database Establishment of *I. Obliquus*

A compound database including 52 compounds (Figure S3) isolated from *I. obliquus* was established by retrieving Internet search engines, such as Chemical Abstracts Service (CAS) database, Web of Science, ChemSpider and literature. They were then energy minimized using CHARMm force filed and thereafter exploited as ligands.

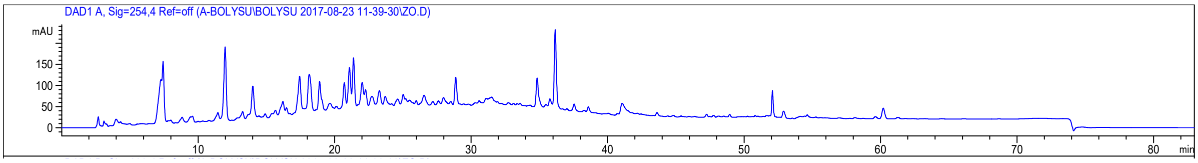

Figure S1. The fingerprint of the ethanol extract of *I. obliquus* (IOE). HPLC conditions-column: Waters Atlantis T3 RP-C18 column, 5 μm, 250 mm × 4.6 mm; the mobile phases: methanol (0.1 % formic acid, A) and water (B); the following elution procedures: 0 min (10% A)→50 min (100% A)→70 min (100% A)→71 min (10% A)→83 min (10% A); flowing rate: 0.8 mL/min; detection wavelength: 260 nm; temperature: 30 °C; injection: 10 μL. Wherein, the peak at 52 min was the one for botulin.

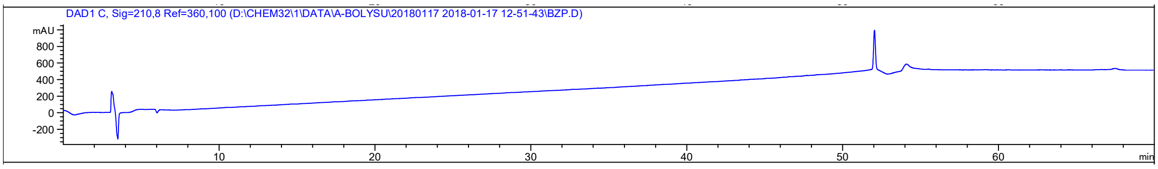

Figure S2. A HPLC chromatogram of the standard chemical compound (betulin) for *I. obliquus* identification. HPLC conditions-column: Waters Atlantis T3 RP-C18 column, 5 μm, 250 mm × 4.6 mm; the mobile phases: methanol (0.1 % formic acid, A) and water (B); the following elution procedures: 0 min (10% A)→50 min (100% A)→70 min (100% A)→71 min (10% A)→83 min (10% A); flowing rate: 0.8 mL/min; detection wavelength: 260 nm; temperature: 30 °C; injection: 10μl. Wherein, the peak at 52 min was the one for botulin.

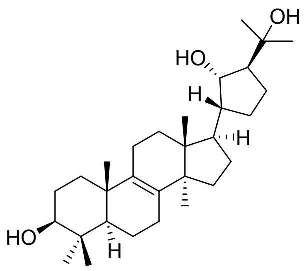

inonotusane A (1)

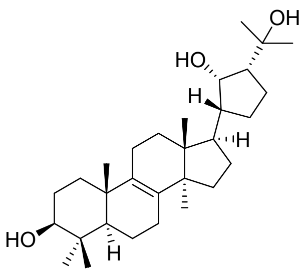

inonotusane B (2)

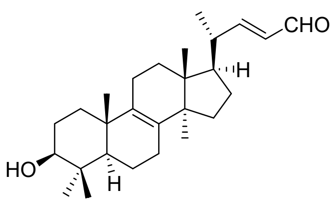

inonotusane C (3)

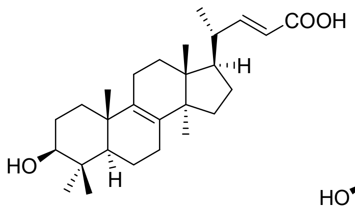

3-beta-hydroxy-25,26,27-trinorlanosta-8,22E-dien-24-oic acid (4)

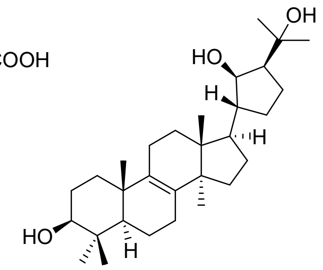

inonotsutriol A (5)

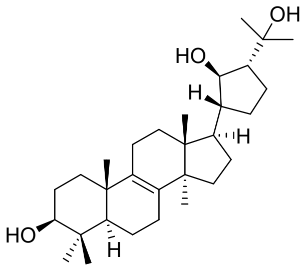

inonotsrtriol B (6)

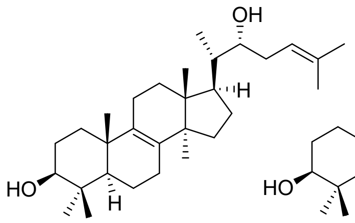

inotodiol (7)

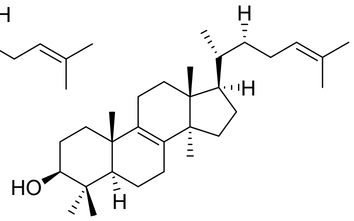

lanosteol (8)

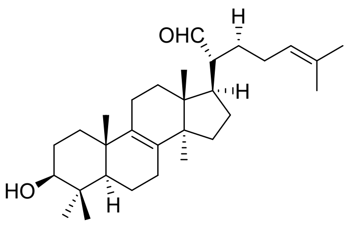

3-beta-hydroxy-lanosta-8,24-diene-21-a (9)

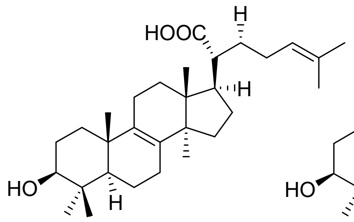

trametenolic acid (10)

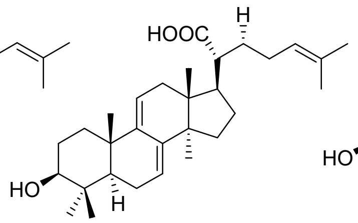

3-beta-hydroxylanosta-7,9(11),24-trien-21-oic acid (11)

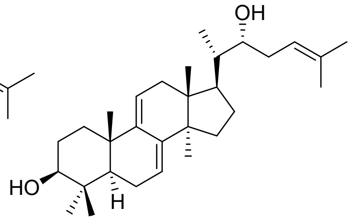

3-beta,22-dihydroxylanosta-7,9(11),24-triene (12)

Figure S3. Structures of compounds collected for database establiment.

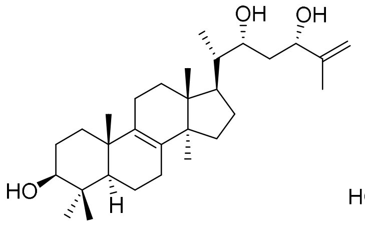

inonotsutriol E (13)

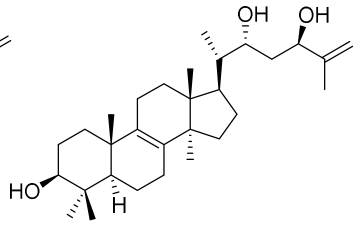

inonotsutriol D (14)

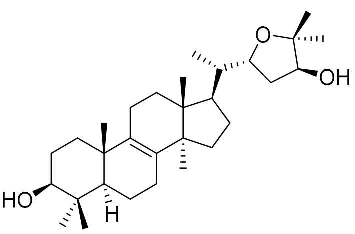

inonotsuoxide A (15)

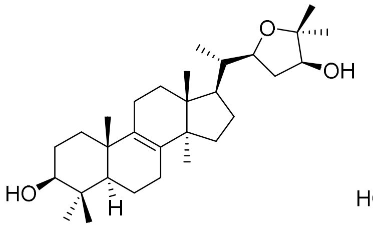

inonotsuoxide B (16)

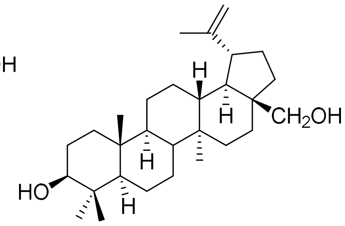

betulin (17)

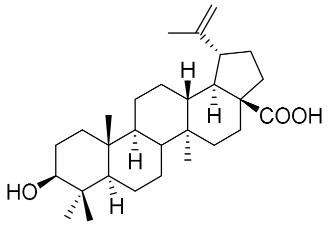

betulinic acid (18)

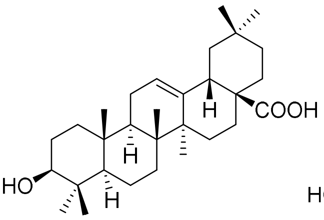

oleanolic acid (19)

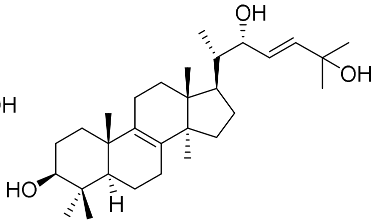

fuscoporianol C (20)

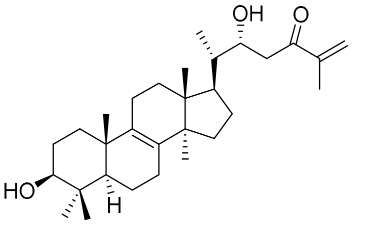

3-beta,22-beta-dihydroxylanosta-8,25-diene-24-one (21)

F

Figure S3. Continued.

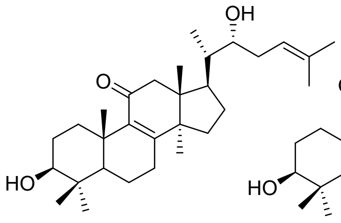

inonotsuoxodiol A (22)

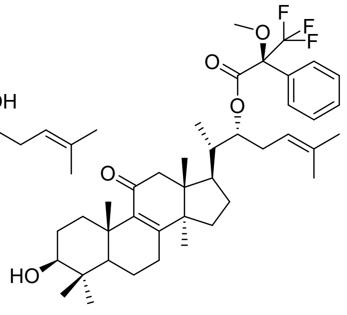

3,22-di-(S)-MTPA ester (23)

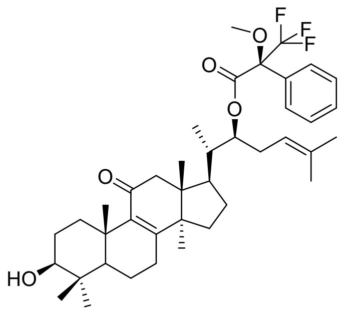

3,22-di-(R)-MTPA ester (24)

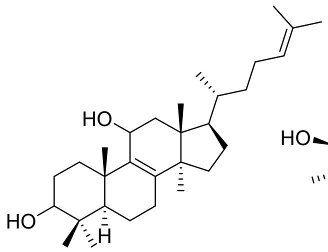

lanosta-8,24-dien-3,11-diol (25)

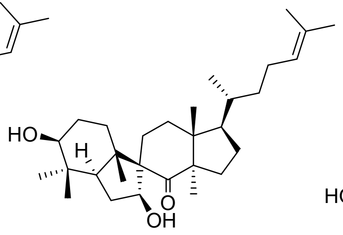

Spiroinonotsuoxodiol (26)

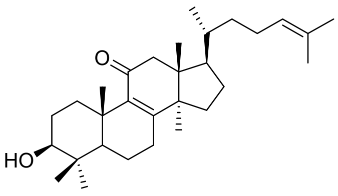

lanosterol (27)

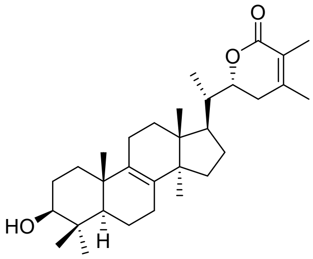

spiroveitchionolide (28)

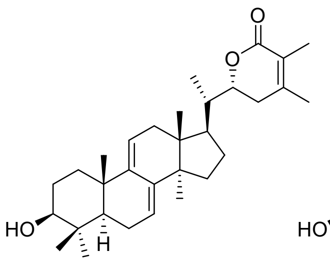

inonotsudiol A (29)

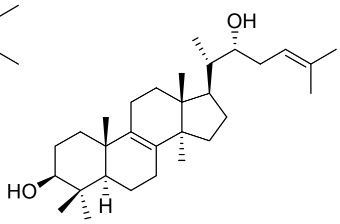

inotodiol (30)

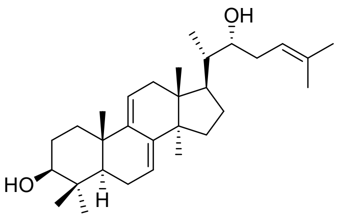

3b,22-dihydroxyanosta-7,9(11),24-triene (31)

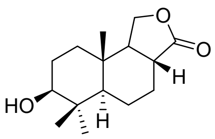

inotolactone C (32)

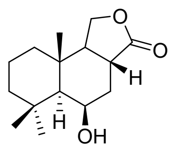

6b-hydroxy-trans-dihydroconfertifolin (33)

Figure S3. Continued.

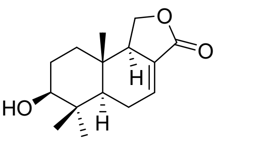

3b-hydroxycinnamolide (34)

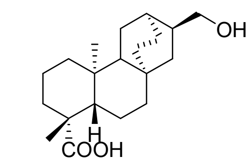

17-hydroxy-ent-atisan-19-oic acid (35)

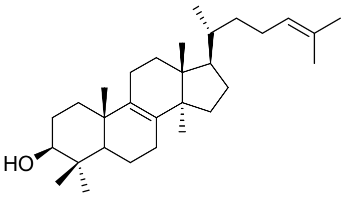

lanosterol (36)

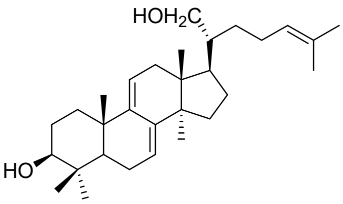

3 -hydroxy-8, 24-dien- 21-al (37)

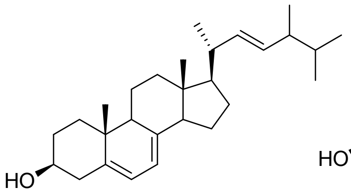

ergosterol (38)

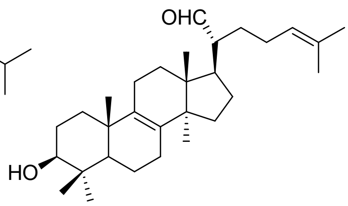

3-beta-hydroxylanosta-8,24-dien-21-al (39)

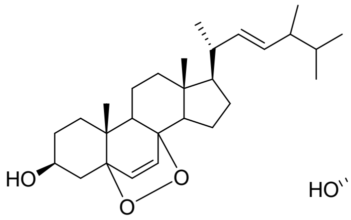

ergosterol peroxide (40)

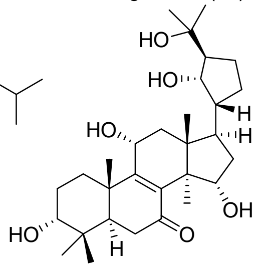

inonotusol A (41)

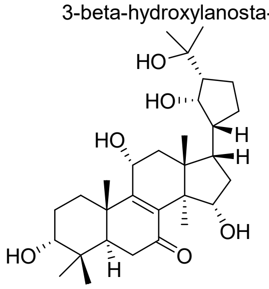

inonotusol B (42)

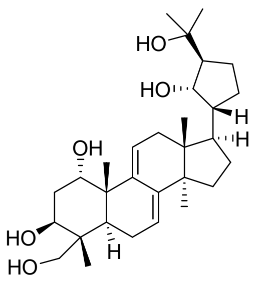

inonotusol C (43)

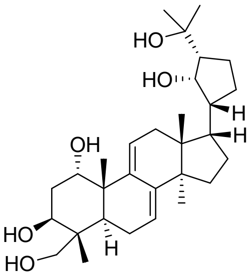

inonotusol D (44)

Figure S3. Continued.

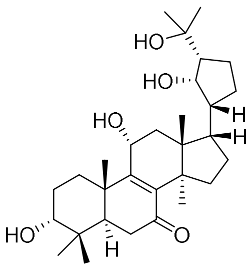

inonotusol E (45)

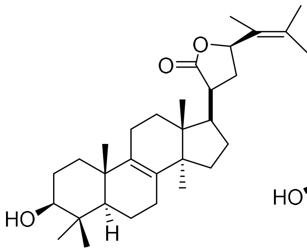

inonotusol F (46)

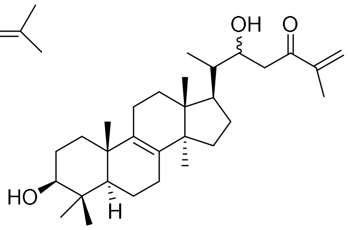

inonotusol G (47)

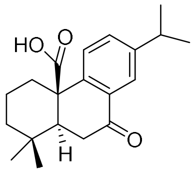

inonotusic acid (8)

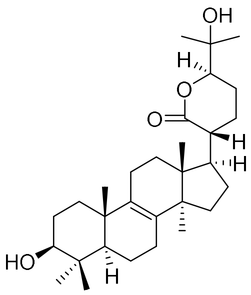

inonotsulide A (49)

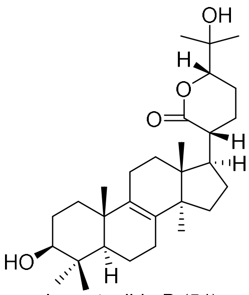

inonotsulide B (51)

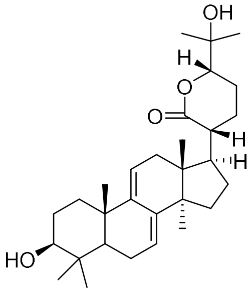

inonotsulide C (52)

Figure S3. Continued.
